# Analysis of volatile compounds in fresh sturgeon with different preservation methods using electronic nose and gas chromatography/mass spectrometry

**DOI:** 10.1039/c9ra06287d

**Published:** 2019-11-28

**Authors:** Wenfu Hou, Qianhui Han, Heng Gong, Wen Liu, Hongxun Wang, Min Zhou, Ting Min, Siyi Pan

**Affiliations:** College of Food Science and Technology, Huazhong Agricultural University 1st Shizishan Road Wuhan Hubei 430070 P. R. China pansiyi303@163.com; College of Food Science and Engineering, Wuhan Polytechnic University Wuhan Hubei 430023 P. R. China; School of Biological and Pharmaceutical Engineering, Wuhan Polytechnic University Wuhan Hubei 430023 P. R. China

## Abstract

Contamination of microorganisms causes a rapid deterioration in the quality of fresh sturgeon meat, which results in the shortening of the shelf-life and increase in the health risk. In this paper, two preservation treatments based on microbial control were considered. During the chilling storage (0–6 days) period, the sensory analysis and the volatile compound (VOC) evaluation were performed by electronic nose and SPME-GC/MS. Results showed that washing with acidic oxidized electrolyzed water and the addition of ε-PL influences the sensitive VOCs of the fresh sturgeon by inhibiting the spoilage of microbes or introducing the chemical agents like free chlorine and reactive oxygen species. Furthermore, GC/MS analysis detected more than 40 kinds of VOCs, mainly aldehydes and ketones, in the fresh sturgeon during the chilling storage period. The relative content of heptanal, nonanal, and acetophenone increased linearly with the storage time in all the groups, where *R*^2^ of all the groups was larger than 0.9. However, the content of hexanal and octanal decreased simultaneously. This indicated that the present work discovered the potential biomarkers acting as indicators for rapidly evaluating the quality of sturgeon products.

## Abstract 2

Contamination of microorganisms caused rapidly quality deterioration of fresh sturgeon meat. During the chilling storage, the sensory analysis and volatile compounds evaluation were well performed by electronic nose and SPME-GC/MS.

## Graphical abstract

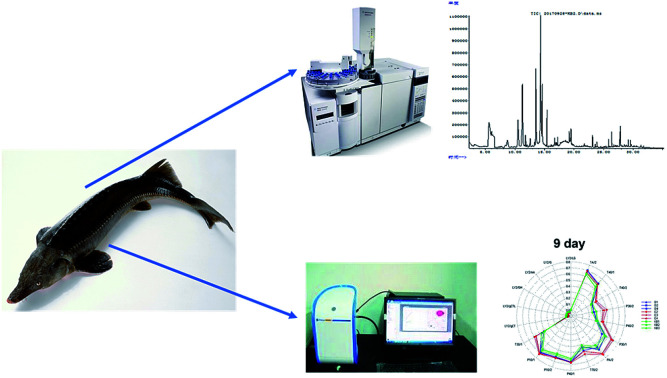

## 1. Introduction

The sturgeon belongs to one of the oldest families of bony fish. It is a native of subtropical, temperate, and sub-Arctic rivers, lakes, and coastlines of Eurasia and North America.^1^ The sturgeon not only has important economic value, such as food, nutrition, nourishment, health care, medicinal, and ornamental, but also has great significance in scientific research.^2^ China's sturgeon resources are very rich, especially the artificial breeding, which feeds the sturgeons with the commercially available diets. Until now, the edible sturgeon products are still dominated by dried, smoked or salted versions. Product development of the fresh sturgeon is less, so the methods for the preservation and quality control of the fresh sturgeon products are urgent problems to be solved.

Living, fresh or refrigerated fishes are the most popular forms for human consumption, accounting for the largest proportion (45%) in 2016. The spoilage of fresh fish after slaughter is mainly dependent on various biochemical reactions (including protein hydrolysis and lipid oxidation by endogenous enzymes) and the growth of bacteria.^3^ Numerous methods like freezing, adding antiseptic substances, and modified atmosphere packaging (MAP) have been focused on to maintain the freshness of the aquatic products by inhibiting the reactions. The acidic electrolyzed oxidizing water (AEOW) is synthesized by electrolyzing the NaCl solution (<0.1 g per 100 mL) in an electrolysis chamber and has been recognized as an environmentally friendly and highly effective antimicrobial agent to reduce the foodborne pathogens on the fresh food.^4,5^ Generally, AEOW has a low pH (<2.8), high oxidation–reduction potential (ORP > 1050 mV), and high available chlorine concentration (ACC is greater than 5 mg L^−1^).^6^ Park *et al.* found that AEOW treatment completely eliminates *Listeria monocytogenes*, *Salmonella*, and *Escherichia coli* (O157 : H7) from lettuce and spinach leaves.^7^ However, AEOW might get altered with the pH or induced in the oxidative substrates. On the other hand, the addition of natural antibacterial compounds does not change the chemical environment of the food. ε-Polylysine (ε-PL) is a natural polypeptide comprising 25–35 l-lysine units.^8^ It has high chemical stability and exhibits wide-spectrum antimicrobial activity against the Gram-positive and Gram-negative bacteria, yeast, and fungi.^9^ No toxicity of ε-PL on rats has been demonstrated,^10^ and it is approved for usage as a food preservative in Japan.

Sensory detection, which is induced by volatile substances generated during the storage, is a direct way to evaluate the quality of the aquatic products.^11^ An electronic nose (enose, EN) and solid phase microextraction-gas chromatography-mass spectrometry (SPME-GC/MS) are effective ways to characterize the changes in the volatile components.^12,13^ The advantages of EN for detecting and distinguishing odors in food are simple operation, good repeatability, and high sensitivity.^14,15^ The EN could be utilized for the early detection of contamination and defects in the foodstuffs.^16^ Wang *et al.* used an EN to predict the total viable counts (TVC) in chilled pork.^17^ In contrast, SPME-GC/MS is a quantitative analytical method,^18^ which has been used to study detailed volatile organic compounds (VOCs) in foods, such as prawns,^19^ crabs,^20^ and fish.^21,22^ Parlapani *et al.* found that some VOCs were associated with the metabolic activity of a particular microbial group, *e.g.*, ethyl esters were linked with *Pseudomonas*, while 2,3-methylbutanal and 3-hydroxy-2-butanone were associated with *Carnobacterium* and *Lactobacillus*. Therefore, GC/MS serves as a possible application for rapid freshness assessment.^23,24^ In this study, both the EN and SPME-GC/MS were combined to detect and analyze the volatile organic compounds (VOCs) of the fresh sturgeon under different processing treatments during storage in the refrigerator. The EN was used to judge the comprehensive sensory changes in the sturgeon products (qualitative analysis), while SPME-GC/MS was employed for the quantitative analysis of the VOCs. Finally, the results obtained could help to assess the freshness of the fresh sturgeon quality with different preservation methods (AEOW and addition of ε-PL), and at the same time, allow to pick the better way for maintaining the quality of fresh sturgeon.

## 2. Materials and methods

### 2.1. Materials

A total of 21 tail sturgeon (*Acipenser gueldenstaedti*, cultured in Qingjiang River, Yichang City, Hubei Province, China), with an average body weight of 1500 ± 300 g, was purchased from the local fisheries market. Chemicals used in the experiments, such as sodium chloride (analytic grade), acidic electrolyzed oxidized water (AEOW), and ε-polylysine (ε-PL), were purchased and used directly without any purification. All the animal procedures were performed in accordance with the Guidelines for Care and Use of Laboratory Animals of Huazhong Agricultural University, and all the experiments were approved by the Animal Ethics Committee of Huazhong Agricultural University (approval ID: SYXK2015-0084).

### 2.2. Sample preparation and preservation treatments

#### 2.2.1. Pre-preparation of the samples

After anesthetizing the sturgeon, they were cleaned and cut into pieces of uniform size (weighing 20–30 g) with a sterilized knife. The moisture on the surface of the fish was removed by a sterile filter paper. Each set of the fresh sturgeon was randomly divided into 16 sample boxes, including the control (4 boxes), AEOW treated (4 boxes), ε-PL treated (4 boxes), and 4 boxes for backup. After sectionalizing, the samples were put into the sterile sample box and placed in the refrigerator at 4 °C. Measurements of EN and GC/MS were performed every three days (day 0, 3, 6, and 9) until the sturgeon was thoroughly deteriorated. The sample was picked when the storage time arrived and did not reuse the sample. The 0 day storage of the control group was regarded as a blank measurement.

#### 2.2.2. Acidic electrolyzed oxidized water treatment (AEOW)

The pre-prepared samples were soaked in AEOW (available chlorine concentration of 70 mg L^−1^, pH 2.3, ORP 1100 mV) with a volume ratio of 1 : 2 (w/v). After 10 min, the pieces of sturgeon were drained and placed in a sterile sample box, and the box was placed in a refrigerator at 4 °C.

#### 2.2.3. Addition of ε-PL

The pre-prepared samples were immersed in a 0.5% ε-PL aqueous solution with a volume ratio of 1 : 3 (w/v) for 3 min. Then, they were drained and placed in a sterile sample box, and the box was placed in a refrigerator at 4 °C.

### 2.3. Test of electronic nose (EN)

In the experiment, each group of the sturgeon samples (4 g) for the EN test was accurately weighed and placed in 10 mL sterile vials suitable for the EN test. The data were detected using a metal oxide semiconductor (MOS) based on the gas analyzer array electronic nose detector combined with a headspace auto-sampler (Alpha M. O. S., FOX 4000, France). The measurement started after 300 s of equilibration at 60 °C under agitation (500 rpm). The injection volume was 3500 μL with an injection speed of 2500 μL s^−1^. The data acquisition lasted for 180 s. Each sample was measured for 4 readings per sample, but the first reading was discarded for the certainty of sensor stabilization. The EN signal response of the samples was calculated using the following expression:*R* = (*R*_0_ − *R*_t_)/*R*_0_where *R* is the EN sensor signal response, *R*_t_ is the value of the conductance of the MOS sensors, and *R*_0_ is the value of MOS sensors at time 0 for each sample. The featured extraction of the signals was obtained when they showed the peak value in the curve of time responses of an array of eighteen gas sensors. For the MOS sensor of type PA/2, P30/1, and P40/1, mainly for aldehydes, the detection range was 0–1000 ppm and the detection limit was 1 ppm. Similarly, for the MOS sensor of type P40/2, PA/2, and LY2/AA, the detection range was 0–1000 ppm and the detection limit was 1 ppm, as well as for P30/2 and P30/1 type MOS sensor, mainly for ketones, the detection range was 0–300 ppm and the detection limit was 1 ppm. Likewise, for the MOS sensor of type P30/1, PA/2, and LY/gCT, mainly for alcohols, the detection range was 0–1000 ppm and the detection limit was 1 ppm. Further, the sensor response data were analyzed by the software Unscrambler X 10.4 (64 bit, CAMO Software Inc. USA). Subsequently, the characteristic value of the optimized sensor response was subjected to the principal component analysis (PCA).

### 2.4. SPME-GC/MS

The sampling protocol was designed as follows: 5 g of each group of the sturgeon samples were weighed accurately. 15 mL of the saturated NaCl solution was then added in a ratio of 1 : 3 (w/v), and was then homogenized. Then, 5 g of the mixtures were placed in 10 mL glass vials; each sample was prepared in triplicate.

VOCs were extracted by SPME (Supelco, Bellefonte, PA, USA) with a coated fiber of PDMS/DVB (polydimethylsiloxane/divinylbenzene, coating thickness was 65 μm). The SPME fiber was exposed to the headspace of the sample for 30 min at 60 °C. The GC-MS analysis was performed by 7890A-5975C (Agilent Technologies Inc., USA) with a DB-5 column (30 m × 0.25 mm, 0.25 μm). The oven temperature was set at 40 °C for 4 min, programmed by an increase in the temperature at a rate of 6 °C min^−1^ to 200 °C for 5 min, and then an increase of 10 °C min^−1^ to 250 °C for 5 min. Helium was employed as the carrier gas at a constant flow rate of 1.0 mL min^−1^ (splitless mode). The temperature of the mass spectrometry source and quadrupole was set at 230 and 150 °C, respectively. All the analyses were performed by setting the ionization energy at 70 eV, with the mass scan range being 50–400 *m*/*z*.

Analytical methods: (1) qualitative analysis: the compounds were searched by computer and matched with NIST11 (107 000 compounds) and Wiley Library (320 000 compounds, Version 6.0), with a matching degree of 80% or more; the literature qualitatively analyzes the substances detected in the experiment. (2) Quantitative analysis: the relative percentage content was calculated by the peak area normalization method. The preliminary experiments were undertaken to test the repeatability of these analytical methods by analyzing the control group 3 times under the proposed conditions. The relative standard deviation (%RSD) presented in the study was in the range of 5–19% with an average value of 9%.

### 2.5. Statistical data analysis

The electronic nose data was collected by the MOS sensor, converted into a data table by the fingerprint analysis, and then, the software Unscrambler X 10.4 (64 bit, CAMO Software Inc. USA) was used for the calculations. Multivariate analysis methods include principal component analysis (PCA) and radar image analysis. PCA was performed using the SPSS 19.0 software, and the data were statistically processed and plotted with Microsoft Excel 2010. The GC-MS data used the NIST 11 library and the Wiley library for the qualitative analysis of volatile components (matching greater than 80, maximum 100). Eventually, the total area of the volatile flavor components was calculated by Microsoft Excel 2010.

## 3. Results and discussion

### 3.1. Sensory evaluations of sturgeon chilling storage under different treatments by EN

**Fig. 1:**
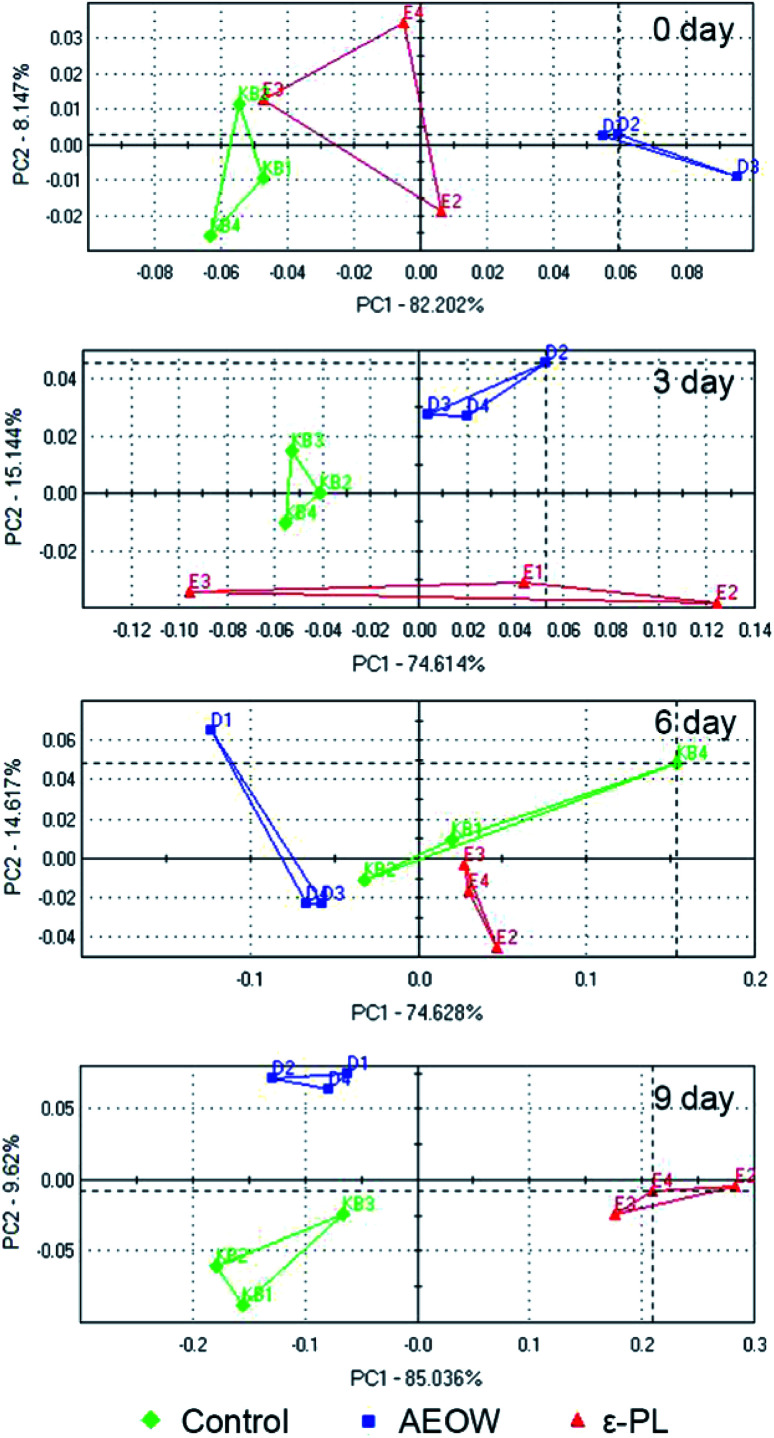
The plot of the first two principal components of the PCA model built with the electronic nose data related to the chilling sturgeon treated with different preservation methods for 0, 3, 6, and 9 days, respectively.

**Fig. 2:**
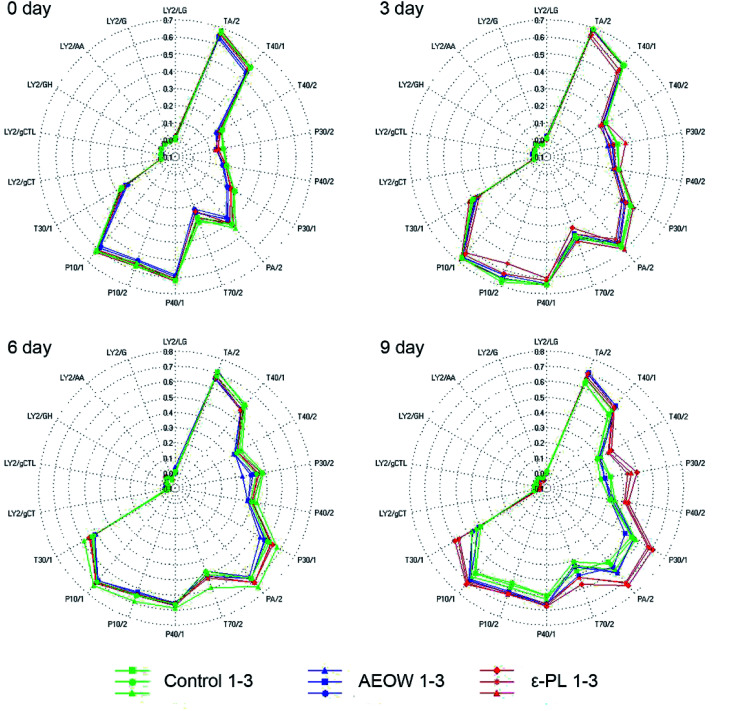
The radical plots of the chilling sturgeon treated with different preservation methods for 0, 3, 6, and 9 days, respectively.

An electronic nose (EN) and electronic tongue^25^ have been designed to simulate human senses of smell and taste in a great possible way.^26^ The principal component analysis (PCA) graph was obtained to identify the patterns of correlation with individual composition variables among the fresh sturgeon during the chilled storage with different treatments (Fig. 1). A sum of the contribution percentages of PC1 and PC2 for all the groups was determined to be above 90%, indicating that PC1 and PC2 contained most of the volatile characters' information. A clearly different distribution of the volatile odor of the sturgeon under different treatments in the PCA graph confirmed that the MOS module has the ability to respond accurately.^27^ According to Fig. 1, all the datasets were located in three non-overlapping regions, indicating that the samples of control, AROW, and ε-PL were easily differentiated. For example, at the initial stage, an obvious separation between the AEOW treatment and the control group occurred along the PC1 (82.20%). At the same time, the spider plots provided a graphical representation of the flavor profiles for different sensory detectors (Fig. 2). The characteristic fingerprint identified by 18 sensors of the initial treatments illustrated that the sensor response at T70/2, PA/2, P30/1, P30/2, TA/2, and T40/1 showed differences. However, in the case of AEOW, the reason for getting combined with the chemical reagent in the treating groups might be attributed to the free chlorine and reactive oxygen species present in AEOW.^28^

From 0 to 9 days, the plots in the PCA chart of these three groups were distributed significantly different with the prolonged storage time. At 0 day storage, all the radial curves of each group were similar, and the signal response intensity for all the 18 attributes was also very close, indicating that the difference in flavorings between the samples was not obvious. Further, the radial plots displayed differences when the sturgeon was stored after 6 d, especially in channel T30/1, P10/2, T70/2, PA/2, P30/1, and PA/2. Particularly, at the 9 day storage, from the visible check of the naked eyes, the control group tended to be spoiled. A separation between the control and ε-PL groups appeared in PC1 (85.04%), whereas the difference with AEOW was observed in PC2 (9.62%). The response strength on the sensor T30/1, T70/2, PA/2, P30/1, P40/2, P30/2, and T40/2 became weak in the control group, indicating that the spoilage might induce the weaker response strength. On the other hand, the radial plot of the control and AEOW groups at the 9 day storage seemed quite similar, and it should be noticed that the distance between these two flavorings was relatively less.^17^ Moreover, it has been demonstrated by the former researches that the EN system cluster analyzed by PCA could be a simple and rapid technique for monitoring the shelf-life of *Tuber magnatum* Pico during the storage,^29^ and thus, it can serve as a specialized gas-sensing instrument for fruit identifications, ripeness assessments *etc.*^30^ Therefore, the EN could be used for the assessment of freshness, thereby detecting the presence of the off-flavor compounds and discriminating different fish species.^31^

### 3.2. Volatile components of sturgeon during different treatments measured by SPME-GC/MS

**Fig. 3:**
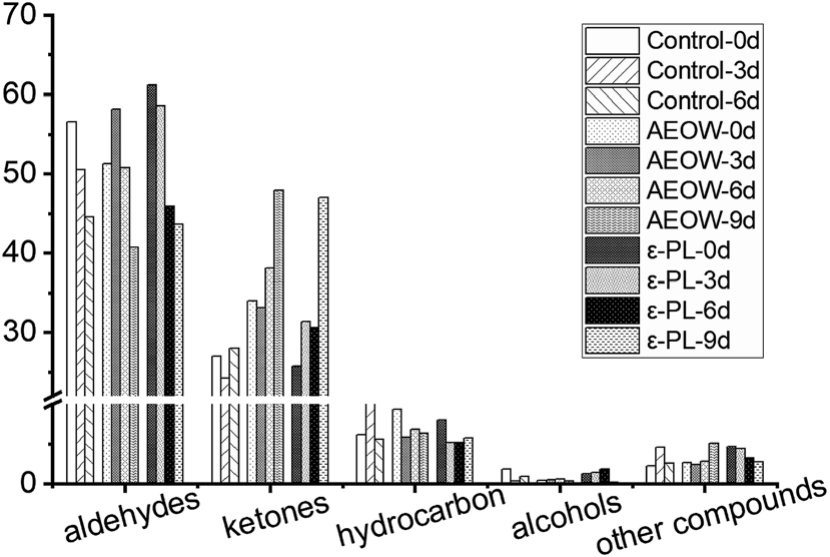
The summary of volatile organic compounds (VOCs) categorized as aldehydes, ketones, hydrocarbon, alcohols, and other compounds of fresh sturgeon with different treatments during chilling storage.

After killing the sturgeon, a strong fishy smell, grassy smell, and fatty scent could be smelled. These odors were mainly attributed to aldehydes, ketones, alcohols, hydrocarbons, and other substances. During the storage, the various biochemical reactions, including degradation of ATP and protein, oxidation of the unsaturated fatty acid, and the growth of bacteria, would generate more volatile substrates to change the smell of the sturgeon products. All of these volatile substrates were named as volatile organic compounds (VOCs). Here, the results showed that more than 40 kinds of VOCs, mainly aldehydes, ketones, alcohols, hydrocarbons, and a small amount of benzene compounds, were characterized by GC/MS (Fig. 3). Among the 40 kinds of VOCs, twelve kinds of VOCs were picked as the key indicators because their changes in the relative content (decrease or increase) varied evidently during the chilling storage period with different preservation treatments. According to the database of mass spectrometry, the 12 kinds of VOCs were identified as hexanal, heptanal, benzaldehyde, octanal, nonanal, decanal, acacia aldehyde, 2,5-dioctylketone, acetophenone, 3,5-octadien-2-one, 5,8-undecadien-2-one, 6,10-dimethyl-*E*, and 1,2-octadecane. The detailed illustrations of the influence of preservation treatments on the VOCs of sturgeon under different storage time are as follows:

#### 3.2.1. Changes of aldehydes during the storage of sturgeon with various preservation treatments

Table 1 shows the aldehyde compounds and their relative percentages during the storage of fresh sturgeon with different treatments. In all volatile compounds, the content of aldehydes was the highest, with the percentage being more than 50%. More than 17 kinds of components were identified as the main volatile component. Many studies have shown that aldehydes play an important role in imparting the flavor to the aquatic products, but the threshold was found to be relatively low.^32^ Aldehydes mainly originate from lipid oxidation. The hexanal odor is described as green, grassy, powerful, and penetrating.^33^ Hexanal may be formed from oleic acid or linoleic acid upon undergoing degradation by linoleate hydroperoxides or maybe by the degradation of other unsaturated aldehydes. Octanal has a fruity aroma and a grassy scent. Heptanal and nonanal are from the oxidation of oleic acid, thereby imparting fishy smell and rancidity, and hence, are one of the main fishy taste substances of the aquatic products. Benzaldehyde is formed by the degradation of linoleic acid, and also involves other non-fatty oxidation pathways like amino acid degradation.^34^ The odor of benzaldehyde has been described as candy, sweet, and almond-type, which is linked to a pleasant almond odor. The higher threshold has a smaller direct effect on the fish odor, but with a certain side effect on the fishy odor.^33^ In addition to their direct contribution to odor, aldehydes can react further with other compounds to produce flavor chemicals, such as formaldehyde, acetaldehyde, and malonaldehyde, which in turn react with hydrogen sulphide, ammonia, and glucose to form a series of heterocyclic compounds.^35^

**Table 1:** The retention time and relative percentages of aldehydes in VOCs of sturgeon with different treatments

Aldehydes	Retention time/min	Relative percentage %										
		Control			AEOW				ε-PL			
		0 d	3 d	6 d	0 d	3 d	6 d	9 d	0 d	3 d	6 d	9 d
Hexanal	5.62	26.72	17.74	15.73	17.76	24.39	21.33	8.13	25.58	23.21	15.16	10.64
Heptanal	8.69	2.92	4.07	6.90	1.20	1.68	3.69	4.44	2.54	3.45	4.60	5.92
Benzaldehyde	10.49	5.20	5.73	6.08	6.46	6.63	7.37	7.07	6.75	6.77	6.86	7.03
Octanal	11.77	6.41	2.78	2.09	4.89	3.06	1.40	1.51	5.44	3.53	1.52	2.07
(*E*,*E*)-2,4-Heptadienal	11.99	1.01	0.35	0.57	0.92	0.80	0.23	0.73	0.88	1.35	1.18	0.81
2-Octenal	13.33	1.17	0.61	0.12	0.58	0.65	0.52	0.61	1.04	1.07	0.77	0.50
Nonanal	14.60	6.39	9.80	11.32	10.85	11.98	12.99	14.09	10.55	12.24	12.49	12.70
2-Hydroxynonenal	16.05	0.29	0.19	0.12	—	0.27	0.11	—	—	0.28	0.14	—
3-Ethylbenzaldehyde	16.14	0.60	0.55	0.26	0.52	0.63	0.47	0.98	0.54	0.71	0.34	0.29
Decanal	17.21	2.07	4.55	0.72	4.50	5.30	1.65	1.89	5.17	3.55	2.14	2.21
2,4-Nonadienal	17.46	0.20	—	—	0.14	0.32	—	—	0.14	0.19	—	—
2-Decenal	18.57	0.30	0.75	0.22	0.42	0.30	0.22	0.22	0.47	0.44	0.16	—
Undecanal	19.65	0.50	0.92	0.16	0.58	0.64	0.22	0.20	0.67	0.48	0.18	0.54
2,4-Decadienal	19.90	0.73	1.12	0.07	0.47	0.35	0.22	0.37	0.41	0.69	0.08	0.19
Dodecanal	21.93	0.28	0.73	0.10	0.40	0.50	0.10	0.2	0.58	0.36	0.10	0.26
Tridecylic aldehyde	24.07	0.16	0.32	—	—	0.30	—	—	—	—	—	0.21
Acacia aldehyde	30.11	1.65	0.31	0.13	1.55	0.36	0.26	0.25	0.43	0.24	0.15	0.22
Sum		56.6	50.52	44.59	51.24	58.16	50.78	40.69	61.19	58.56	45.87	43.59

In the initial stage of treatment (0 day), the content of hexanal, heptanal, and nonanal for the control and ε-PL addition groups had a similar value. Nevertheless, the AEOW wash directly decreased them, indicating that AEOW wash would decrease the grassy odor of the fresh sturgeon. On the other hand, the immediate changes in the flavor components were attributed to the strong odor of the acidic electrolyzed water itself. Upon increasing the storage time (3–9 days), the relative percentage of hexanal and octanal in the samples of each group generally showed a downward trend with varying degrees. The reason might be the inhibition of the degradation of oleic acid by the preservation method. The octanal had a good effect on the flavor (aroma component) of sturgeon. The proportion of octanal decreased in the samples during the shelf life of each group (the control, AEOW group, and addition of ε-PL group), and the retention rates were determined to be 32.61%, 30.88%, and 38.05%, respectively. Thus, the results showed that the addition of ε-PL would help in maintaining the aroma flavor of sturgeon.

The relative percentages of heptanal, nonanal, and benzaldehyde exhibited an upward trend during the chilling storage, resulting from the deep oxidation of unsaturated lipids, such as oleic acid, linoleic acid, and arachidonic acid. The preservation treatments inhibited the generation of heptanal effectively, thereby revealing that they assist in lowering the oxidative degradation of fatty acids. Other unsaturated aldehydes also contributed to the comprehensive flavor of the sturgeon, like 2,4-decadienal, 2,4-heptane aldehyde, 2-octenal aldehyde, and 2-decenal. They all belonged to the linoleic acid degradation products. 2-Nonenal is a degradation product of oleic acid and linoleic acid, often with oily rancid and fishy smell. Further, 2,4-heptanedialdehyde has a grass odor, while 2,4-nonadienal has a fatty taste.^36^

#### 3.2.2. Changes of ketones during the storage of sturgeon with various preservation treatments

GC/MS detected only 4 kinds of ketones (2,5-dioctylketone, acetophenone, 3,5-octadien-2-one, and 5,8-undecadien-2-one, 6,10-dimethyl-*E*), but the concentration of these ketones was second only to aldehydes. Ketones are mainly produced by the oxidation of unsaturated fatty acids, microbial metabolization or amino acid degradation.^37^ Generally, ketones have higher threshold values and thus, contribute less to odor, but act as impactful odorants in the flavor of the sturgeon meat. Among the 4 ketones, 2,5-dioctylketone was abundant. According to the former studies, 2,5-octanedione has a relatively large effect on the fishy smell, thereby displaying a discordant metallic taste.^38^

The data presented in Table 2 showed that the relative percentage of 2,5-octanedione in each group of sturgeon increased significantly during the storage time. It might be caused by the oxidation of fatty acids and the microbial growth and reproduction. The increase in the 2,5-octanedione content in the AEOW group and the ε-PL addition group was found to be 70.28% and 61.72%, respectively. For the AEOW group, the free radical in the AEOW could promote the oxidation of lipids and generate more 2,5-octanedione. Apart from 2,5-octanedione, the content of acetophenone was also relatively increased. Moreover, the studies showed that acetophenone had a mushroom odor, but due to the high threshold, acetophenone had a little effect on the odor characteristics of the fish. A degradation product of *n*-3 polyunsaturated fatty acids, 3,5-octadiene-2-one, which was observed in the volatile fraction of fresh oysters, decreased with an increase in the storage time.^39^

**Table 2:** The retention time and relative percentages of ketones and hydrocarbon compounds in VOCs of sturgeon with different treatments

Compounds	Retention time/min	Relative percentage %										
		Control			AEOW				ε-PL			
		0 d	3 d	6 d	0 d	3 d	6 d	9 d	0 d	3 d	6 d	9 d
**Ketones**												
2,5-Dioctylketone	11.26	14.86	15.78	15.48	16.59	20.00	22.69	28.25	16.85	18.16	14.15	27.25
Acetophenone	13.52	4.51	6.82	11.35	7.22	5.42	12.02	16.84	3.13	4.55	13.03	16.42
3,5-Octadien-2-one	13.63	6.93	1.06	1.10	9.10	7.24	3.22	2.40	5.20	8.50	3.30	3.05
5,8-Undecadien-2-one, 6,10-dimethyl-, (*E*)-	22.73	0.68	0.59	0.11	1.09	0.48	0.14	0.39	0.59	0.14	0.14	0.22
Sum		26.98	24.25	28.04	34.00	33.14	38.07	47.88	25.77	31.35	30.62	46.94

**Hydrocarbon**												
1-Ethylene cyclohexene	11.61	0.73	—	—	0.64	—	—	—	0.47	0.55	—	—
1,4-Cyclooctadiene	16.97	0.46	0.59	0.20	0.50	0.50	0.17	—	—	0.33	0.11	0.22
Tetradecane	21.73	0.46	1.50	0.19	0.58	0.59	0.18	0.13	0.40	0.35	0.63	0.50
Octacosane	23.58	0.72	2.10	0.36	1.13	0.51	0.11	0.60	1.11	0.17	0.38	0.18
Pentadecane	23.79	0.58	2.53	0.45	0.72	0.77	0.48	1.60	0.62	0.54	0.87	0.81
Nonadecane	24.51	0.25	0.66	0.15	0.62	0.18	0.07	0.24	0.55	0.09	0.07	—
Cyclopentadecane	24.93	0.10	0.19	0.26	0.34	0.22	0.46	0.18	0.30	0.16	0.22	0.22
Hexadecane	25.85	0.53	0.96	0.58	0.89	0.53	0.99	0.53	0.52	0.38	0.24	1.07
1,2-Octadecane	26.10	0.36	0.10	—	0.24	0.67	—	—	0.62	0.40	—	—
Heptadecane	27.76	0.61	0.68	1.08	1.50	0.74	1.40	0.85	1.66	0.71	0.65	1.17
2,6,10,14-Tetramethylpentadecane	27.83	0.91	0.63	1.56	1.89	0.96	2.69	1.95	1.51	1.10	1.66	1.27
Octadecane	29.56	0.11	0.16	0.74	0.25	0.15	0.25	0.23	0.23	0.13	0.28	0.23
Sum		6.14	10.31	5.57	9.30	5.82	6.80	6.31	7.99	5.13	5.11	5.67

#### 3.2.3. Changes of hydrocarbon compounds during the storage of sturgeon with various preservation treatments

Upon analyzing the hydrocarbons in VOCs, 11 compounds were detected by GC/MS: 1-ethylene cyclohexene, 1,4-cyclooctadiene, tetradecane, octacosane, pentadecane, nonadecane, cyclopentadecane, hexadecane, heptadecane, 2,6,10,14-tetramethylpentadecane, and octadecane (Table 2). Numerous studies have shown that hydrocarbons are usually found in the detection of volatile substances in the fish species. However, the relative content of the hydrocarbons was found to be quiet low (most of the compounds were below 1%). Furthermore, they have a high threshold, due to which they do not contribute much to the flavor characteristics of the fish. Hydrocarbons are potential contributors to the fishy smell. They endow the products with unpleasant flavor by forming carbonyl compounds under certain circumstances. Among the alkanes, most of them belong to the long linear hydrocarbon compound category, such as tetradecane, pentadecane, hexadecane, heptadecane, octadecane, nonadecane, and octacosane. These linear hydrocarbons generally have a mild odor, resulting from the homogenized degradation of the alkoxy radicals of fatty acid.^40^

With the extension of storage time, then among all the hydrocarbon compounds, 2,6,10,14-tetramethylpentadecane, which smells fresh and sweet, was found to exist in the highest concentration. On the 6th day, the relative percentage of 2,6,10,14-tetramethylpentadecane in each group was found to increase, which might be caused by an increase in the lipid oxidation and carotenoid decomposition. Until the 9th day, the relative percentage of 2,6,10,14-tetramethylpentadecane reduced, indicating that the sturgeon was deteriorated.

#### 3.2.4. Changes of alcohols and other compounds during the storage of sturgeon with various preservation treatments

Alcohols are generally minor contributors to flavor unless present in relatively high concentrations or if unsaturated.^35,41^ In the present work, four alcohols were detected, namely, 3,6-nonadiene-1-ol, eucalyptol, 3,5-octadoene-2-ol, and 2-octen-1-ol (Table 3). The alcohol content was found to be lower than that of aldehydes and ketones, but the thresholds of alcohol were higher than that of aldehydes and ketones. 2-Octen-1-ol is associated with green flavor notes and was identified as the product of the 12-lipoxygenase activity on ω-6 PUFA, such as arachidonic acid in fish tissues.^42^ During the whole storage period, the concentration of alcohols was still less, due to which their contribution to the flavor was limited.

**Table 3:** The retention time and relative percentages of alcohols and other compounds of sturgeon with different treatments

Compounds	Retention time/min	Relative percentage %										
		Control			AEOW				ε-PL			
		0 d	3 d	6 d	0 d	3 d	6 d	9 d	0 d	3 d	6 d	9 d
**Alcohols**												
3,6-Nonadiene-1-ol	12.52	0.98	0.18	0.22	0.18	0.19	0.19	0.36	1.03	1.14	0.31	0.19
Eucalyptol	12.57	—	—	0.66	—	—	0.39	—	—	—	1.55	—
3,5-Octadoene-2-ol	12.74	0.34	0.18	—	0.20	0.33	—	—	0.23	0.28	—	—
2-Octen-1-ol	16.31	0.54	—	—	—	—	—	—	—	—	—	—
Sum		1.86	0.36	0.88	0.38	0.52	0.58	0.36	1.26	1.42	1.86	0.19

**Other compounds**												
Naphthalene	16.72	0.50	1.09	0.92	0.64	0.77	1.33	1.88	0.76	0.92	1.03	1.78
2,3,5,6-Tetramethylphenol	17.56	0.12	0.14	0.36	0.11	0.13	—	—	0.14	0.11	—	—
Methoxybenzene	19.18	0.52	1.94	1.11	0.89	0.63	0.84	1.81	0.15	0.83	0.56	0.32
2,6-Butylated hydroxytoluene	23.92	0.3	0.38	—	0.19	0.37	0.31	1.17	0.95	0.67	1.50	0.40
Isobutyl phthalate	30.57	0.78	0.41	0.15	0.78	0.48	0.25	0.22	2.51	1.86	0.13	0.18
Sum		2.22	4.50	2.54	2.61	2.38	2.73	5.08	4.66	4.39	3.22	2.68

In addition to the above-mentioned main types of volatile components, there were some other kinds of VOCs like benzenes, phenols, and lipids (Table 3). Naphthalene is a diphenyl ring compound and has been reported to be responsible for an unpleasant smell of fish. Naphthalene is always detected in lobsters, scallops, and crabs. However, it is generally believed that naphthalene has a great relationship with environmental contaminants.^43^ In this experiment, 2,6-butylated hydroxytoluene was detected, which has been reported in the VOCs of herbs and spices. Ester compounds, like isooctyl phthalate, are generally condensed by the esterification of acid and alcohol and are an important source of meat flavor characteristics. The relative content of other components was generally less throughout the shelf life.

### 3.3. Analysis of critical VOCs content for quality evaluation

Lots of work offer EN as a potential tool in multisensory systems for spoilage examination in foods.^44^ Changes in the generated fingerprint, which result from the metabolism of spoilage bacteria, includes the appearance of new chemical compounds or variations in the quantity of certain volatile compound. So, the EN provides a simple and convenient method for detecting the origin of food contaminants like microbiological, chemical or physical. However, EN and GC/MS have several differences regarding reproducibility, quantitative analysis, and real-time analysis. The reproducibility of EN depends on the structure and training data set.

**Fig. 4:**
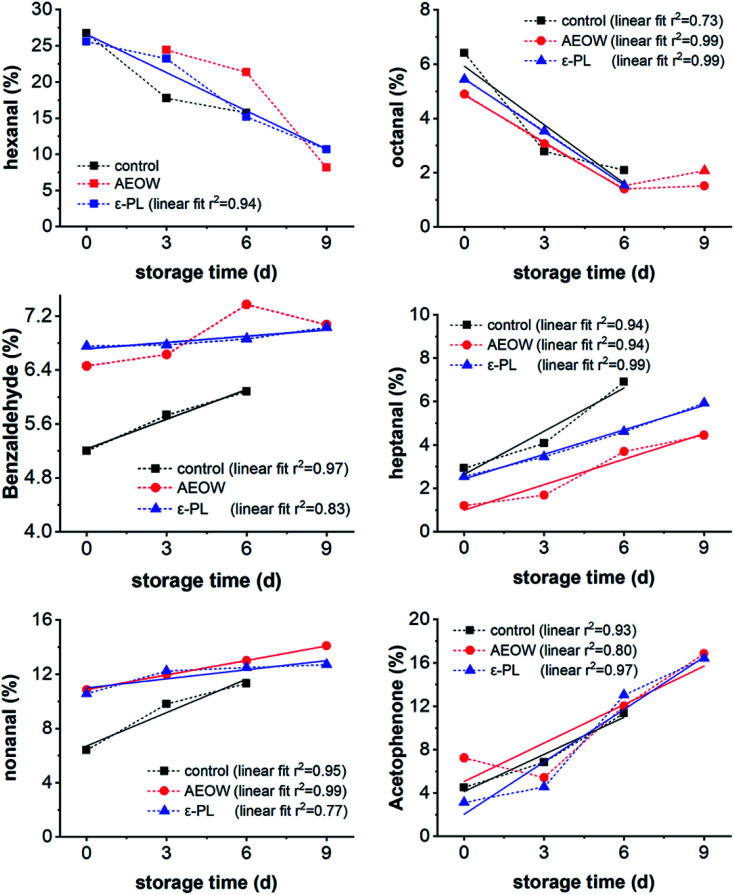
Changes of hexanal, octanal, benzaldehyde, heptanal, nonanal, and acetophenone for fresh sturgeon *vs.* the chilling storage time.

Researchers reported that the type and content of polyunsaturated lipids in various aquatic products are highly dependent on the origin, zone of culture, and processing or preservation treatments, with the detailed categories of VOCs of the aquatic products showing some differences.^32,43^ Upon analyzing all the VOCs in the sturgeon products during the chilling storage with different preservation treatments, 12 kinds of VOCs were picked for correlated fitting. It was found that six of them showed a strong and simple relationship with the storage time (Fig. 4). Further, the data of the control group were collected within 6 days because after 6 days, the sturgeon was totally spoiled and lost the edible characteristics. Moreover, the content of hexanal and octanal decreased with increasing storage time. Within 0–6 days, the linear relationship between the content of octanal and the storage days was clear. However, as the number of days continued to increase, the content of octanal reached a certain low level without any obvious change. Since octanal has a fruity aroma and grassy scent, the spoilage of the sturgeon meat would result in the loss of this flavor. To fit the trends by linear fitting, it was found that the *R*^2^ reached 0.9 or more, indicating that the content of heptanal (*R*^2^: 0.94–0.99), nonanal (*R*^2^: 0.77–0.99), and acetophenone (*R*^2^: 0.80–0.97) showed a linear correlation with the storage time. Therefore, these 3 kinds of compounds were used to quickly determine the quality change of sturgeon.

## 4. Conclusion

In the traditional method, the microbial test and training of the expert team for identifying the VOCs for food spoilage detection is complex and time-consuming.^26^ Further, the determination of the microbiological status, using the biochemical tests, relies mainly on the total viable counts (TVCs) and phenotyping microbial isolates. These methods sometimes provide limited information.^45^ Additionally, the chemical methods can also be utilized to detect the microbial contamination of the food based on the analysis of certain chemical markers. The amounts of total volatile basic nitrogen (TVBN) and trimethylamine can be indicative of fish spoilage,^46^ but these markers increase in fish only during the late stages of storage.^47^

GC/MS focuses on the quantitative analysis of VOCs instead of the EN, but the EN would finish the analysis in real-time. So, we combined both of them together. The EN successfully distinguished the flavor characteristics of the sturgeon treated with different preservation methods during the chilling storage. A total of 40 substances were detected by GC/MS, with these substances mainly belonging to aldehydes, alcohols, ketones, and hydrocarbons. During the whole storage period, the relative content of aldehydes in each sample continued to decrease, but the content of ketones increased. VOCs were commonly generated from the degradation catalyzed by the endogenous enzymes (like lipoxygenase, lipase enzymes), or due to the growth of bacteria. AEOW and the addition of ε-PL exerted an influence on the VOCs of the sturgeon by at least two ways: (1) inhibiting the spoilage of microbes and (2) giving rise to the odors of added chemical agents like free chlorine and reactive oxygen species. The content of heptanal, nonanal, and acetophenone increased linearly with the storage time in all the groups, but the content of hexanal and octanal decreased. Thus, the present work provides the potential biomarkers for sensory and qualitative evaluation during the commercial sturgeon storage.

## Abbreviations used

SPME: Solid-phase microextraction
GC-MS: Gas chromatography/mass spectrometry
EN: Electronic nose
VOCs: Volatile organic compounds
ε-PL: ε-Polylysine
AEOW: Acidic electrolyzed oxidizing water
ORP: Oxidation reduction potential
MOS: Metal oxide semiconductor
PCA: Principal component analysis
MAP: Modified atmosphere packaging

## Conflicts of interest

There are no conflicts to declare.

## Supplementary Material
